# Later Meal and Sleep Timing Predicts Higher Percent Body Fat

**DOI:** 10.3390/nu13010073

**Published:** 2020-12-29

**Authors:** Elizabeth A. Thomas, Adnin Zaman, Marc-Andre Cornier, Victoria A. Catenacci, Emma J. Tussey, Laura Grau, Jaron Arbet, Josiane L. Broussard, Corey A. Rynders

**Affiliations:** 1 Division of Endocrinology, Metabolism and Diabetes, Department of Medicine, University of Colorado Anschutz Medical Campus, Aurora, CO 80045, USA; adnin.zaman@cuanschutz.edu (A.Z.); marc.cornier@cuanschutz.edu (M.-A.C.); vicki.catenacci@cuanschutz.edu (V.A.C.); josiane.broussard@cuanschutz.edu (J.L.B.); 2 Anschutz Health & Wellness Center, University of Colorado Anschutz Medical Campus, Aurora, CO 80045, USA; 3 Rocky Mountain Regional Veterans Administration Medical Center, Aurora, CO 80045, USA; 4 Division of Geriatric Medicine, Department of Medicine, University of Colorado Anschutz Medical Campus, Aurora, CO 80045, USA; emma.tussey@cuanschutz.edu (E.J.T.); corey.rynders@cuanschutz.edu (C.A.R.); 5 Department of Biostatistics and Informatics, Colorado School of Public Health, University of Colorado Anschutz Medical Campus, Aurora, CO 80045, USA; laura.grau@cuanschtuz.edu (L.G.); jaron.arbet@cuanschutz.edu (J.A.); 6 Department of Health and Exercise Science, Colorado State University, Fort Collins, CO 80521, USA

**Keywords:** meal timing, sleep timing, time-restricted eating

## Abstract

Accumulating evidence suggests that later timing of energy intake (EI) is associated with increased risk of obesity. In this study, 83 individuals with overweight and obesity underwent assessment of a 7-day period of data collection, including measures of body weight and body composition (DXA) and 24-h measures of EI (photographic food records), sleep (actigraphy), and physical activity (PA, activity monitors) for 7 days. Relationships between body mass index (BMI) and percent body fat (DXA) with meal timing, sleep, and PA were examined. For every 1 h later start of eating, there was a 1.25 (95% CI: 0.60, 1.91) unit increase in percent body fat (False Discovery Rate (FDR) adjusted *p* value = 0.010). For every 1 h later midpoint of the eating window, there was a 1.35 (95% CI: 0.51, 2.19) unit increase in percent body fat (FDR *p* value = 0.029). For every 1 h increase in the end of the sleep period, there was a 1.64 (95% CI: 0.56, 2.72) unit increase in percent body fat (FDR *p* value = 0.044). Later meal and sleep timing were also associated with lower PA levels. In summary, later timing of EI and sleep are associated with higher body fat and lower levels of PA in people with overweight and obesity.

## 1. Introduction

Circadian timing of daily behaviors such as energy intake (EI), sleep and physical activity (PA), has emerged as a key factor in the regulation of body weight [1,2,3]. The internal circadian timing system in mammals is entrained to a 24-h cycle through light signals reaching a master clock in the hypothalamus [4] and has evolved to align activities and behaviors (wake, physical activity and eating, versus sleep, inactivity and fasting) with the day/night cycle [5]. Because metabolic processes are entrained to circadian rhythms, food intake at inappropriate circadian times may lead to adverse metabolic outcomes, including the development of obesity, type 2 diabetes, and cardiovascular disease [6,7].

Dietary patterns have changed over recent decades, with a shift toward more frequent eating events over a longer period of time throughout the day [8,9]. Findings from a number of epidemiological and observational studies reported associations between breakfast skipping and obesity, type 2 diabetes, and cardiovascular disease [10,11,12,13,14,15]. Night shift work is associated with a later and more prolonged eating window [16,17] and food intake at night is linked to obesity despite similar total daily EI [18,19,20,21]. Some reports suggest lower dietary quality in shift workers [17,18,22], while others do not show a difference [23,24,25]. In addition, a higher percent of total daily EI consumed during the evening and night has been associated with greater risk of overweight and obesity [26,27]. Moreover, in two studies examining timing of EI in individuals participating in weight loss interventions, participants who consumed more calories in the morning as compared to the evening lost more weight, despite similar self-reported EI and PA [28,29]. Thus, there is considerable evidence to suggest that inappropriate timing of EI plays an important role in the risk for obesity.

Concurrent with the shift toward a longer daily duration of eating, there is also evidence of reduced sleep duration and quality over the last several decades [30,31,32]. Prospective studies have found associations between short sleep duration and increased risk of obesity, diabetes, hypertension, cardiovascular disease, and weight gain over time [33,34,35,36]. Sleep timing has been less well studied, but reports from some studies have shown associations between later chronotype (i.e., the propensity of an individual to sleep at a particular time of day) and higher body mass index (BMI) and poor dietary quality [3,25,37]. While accumulating evidence suggests associations between later meal and sleep timing and increased risk of obesity, the majority of these studies are based on self-reported rather than objectively measured exposures and outcomes.

Thus, the goal of this study was to evaluate associations between objectively measured free-living meal and sleep timing and BMI, body fat, and variables implicated in body weight regulation, including EI, dietary quality, PA, and sedentary behavior in adults with overweight or obesity. We hypothesized that later timing of both food intake and sleep would be associated with higher BMI and body fat, as well as with poor dietary quality and reduced PA.

## 2. Materials and Methods

Participants: Healthy adults aged 18–50 years with a BMI of 27–45 kg/m^2^ and weight stable (≤5% change over the previous 6 months) with a self-reported typical eating duration >12 h per day were recruited for a behavioral weight loss trial. Participants were excluded for history of cardiovascular disease, diabetes, uncontrolled hypertension, untreated thyroid, renal, hepatic diseases, dyslipidemia, any other medical condition affecting weight or lipid metabolism, night shift work over the previous 6 months, night eating syndrome, or binge eating behaviors. Women who were pregnant, breastfeeding or planning to become pregnant were also excluded. Data from a 7-day baseline assessment period are presented here. For reasons related to feasibility, the 7-day measurements were not performed in a consistent phase of the menstrual cycle for women. The Colorado Multiple Institutional Review Board approved the study protocol and all participants provided written informed consent prior to participation in study-associated procedures. This study was conducted in accordance with the principles expressed in the Declaration of Helsinki. Clinicaltrials.gov NCT03571048.

### Study Design

Screening: Following written informed consent, each participant underwent a history and physical exam with the study physician and completed screening laboratory evaluations including Hemoglobin A1C, lipid panel, comprehensive metabolic panel, TSH, and pregnancy test.

Anthropometrics: Height (without shoes to the nearest cm using a stadiometer) was measured once at baseline. Fasted morning weight (in light clothing) was measured to the nearest 0.1 kg using a digital scale and assessment of body composition (total body mass, fat mass, percent fat) was performed via dual energy x-ray absorptiometry (DEXA; Hologic Discovery W, Bedford, MA, USA).

Meal timing: Photographic food records were used to determine meal timing. Participants used personal smartphones to take pictures of all calorie-containing food and beverages consumed during the 7-day period. A picture of the selected meal was taken before eating and a second picture was taken at the end of the eating period. Participants were provided with detailed instructions regarding methods of taking food photographs (the serving plate occupied the entire field of view, and photographs were taken at a 45° angle so that the depth of foods could be estimated) [38]. After taking the photographs, participants texted photographs directly to a secure account that was monitored in real time by study personnel. Participants were given instructions to prioritize accurate documentation of the timing of the first and last eating occasions of each day. The timestamps on the photographs were used to determine meal timing and eating window. For the purposes of meal timing and EI quantification, days were considered to start at 4 a.m. and to include the following 24 h through 4 a.m. on the next day, such that food eaten between 12–4 a.m. was included within the prior day’s eating window [9]. Total duration of the eating window was calculated based on the start time of the first eating episode through the start time of the last eating episode, with eating episodes defined as food or drink containing any amount of calories. The midpoint of the eating window was the clock time of the halfway point between the start and end of the total the eating window.

Energy Intake: Photographic food records from a consecutive 3-day period (including 2 weekdays and 1 weekend day) were used to estimate EI. The 3 days that were chosen to be used for EI quantification out of the 7-day data collection were pre-specified based on the timing of the placement of sleep and activity monitors. For example, if devices were placed on Friday, the data collection period started at 4 a.m. on Saturday, and the 3-day EI quantification period included Sunday, Monday and Tuesday, whereas if they were placed on a Monday, the data collection period started at 4 a.m. on Tuesday and the 3-day period included Friday, Saturday and Sunday. If the devices were placed in the middle of the week, the 3-day EI quantification period was chosen randomly. A trained registered dietician used these pictures to estimate portion sizes, using the Portion Photos of Popular Foods guide [39]. Dietary intake data were collected and analyzed using Nutrition Data System for Research (NDS-R) software (version 2019), developed by the Nutrition Coordinating Center (NCC), University of Minnesota, Minneapolis, MN [40]. Plausibility of reported EI levels was evaluated by comparing the intake values from the photographic food records with predicted energy expenditure as previously described by Huang et al. [41]. Briefly, plausible data were determined by comparing the regression slope of reported intake on body weight with that of estimated energy expenditure on body weight across a range of cut-off values from ±1 SD to ±2 SD in 0.1 SD increments. Energy expenditure was estimated from an accelerometer worn on the thigh (described below). The optimal cut-off (±1.6 SD) was selected as the first SD value in which the slope of the relationship between intake and body weight was not significantly different from the slope of the relationship between estimated energy expenditure and body weight. The cut-off applied to our data was similar to the value of ±1.4 SD reported by Huang et al. [41]. Participants with 3-day mean EI (kcal/day) lower or higher than the cut-off (±1.6 SD) were excluded. A Healthy Eating Index score was determined based on the 2015 Healthy Eating Index, as previously described [42].

Physical Activity: Free living PA was measured over 7 days using the ActivPAL 3 Micro accelerometer/inclinometer (PALTechnologies, Glasgow, Scotland). The ActivPAL3 micro was placed on the anterior portion of the participant’s non-dominant thigh and uses accelerometer-derived information about thigh position to estimate time spent in different body positions. The time-stamped “event” data file from the ActivPAL software was used to determine time spent sitting, standing, and stepping per day. Daily energy expenditure was determined from the accelerometer data and was expressed in metabolic equivalents per hour (MET-h).

Sleep Patterns: Each subject kept a written log of sleep/wake times and wore a wrist actigraphy and light exposure recorder (Actiwatch Spectrum, Philips Respironics, Bend, OR, USA) to document free-living sleep/wake patterns for 7 continuous days. Rest intervals (i.e., time in bed) were determined using a standardized hierarchical approach described by Patel et al. [43]. Briefly, rest intervals were selected based on the combination of an event marker on the watch, a written sleep diary, a light sensor on the data, and activity data obtained by the watch. Sleep/wake status was then determined from a validated algorithm (Actiware version 6.0.9, Philips Respironics, Bend, OR, USA).

Statistical Analysis: Meal timing, sleep, and PA variables were measured daily for 7 days and then averaged across the week for each subject. EI was measured over 3 days and then averaged for the 3-day period for each subject, and subjects with 3-day mean EI lower or higher than the cut-off (±1.6 SD) were excluded for EI quantification only. “Regularity” was assessed for several variables (meal timing, sleep duration), by calculating the standard deviation (“SD”) of a subject’s daily measures. Descriptive sample statistics (mean, standard deviation) were calculated for all variables. For meal timing and sleep variables, descriptive statistics were calculated for the entire week and stratified by weekdays and weekend days. A Pearson’s correlation heatmap was used to explore relationships between all types of variables. Hierarchical clustering was used to sort the variable names, such that the closer the variable names are listed together, the more positively correlated they are, while variables that are further apart are more likely to have low or negative correlation. Given the exploratory nature of the heatmap, no multiple testing correction was applied. The further apart the variable names are, the more uncorrelated or negatively correlated the variables are.

Univariate linear regressions were used to estimate the relationships between outcomes of BMI and percent body fat with measures of EI, sleep, meal timing, and PA. Age and sex were tested as potential confounders. Measures of EI were included in the linear regression analyses and Pearson’s correlation heatmap; however, they were excluded from the elastic net regression multivariable model due to the high amount of missingness (45.8%) after excluding reported intakes that were deemed implausible. The significance level α = 0.05 was used in all analyses. Both the original *p*-values and False Discovery Rate (FDR) adjusted *p*-values [44] are reported.

Elastic net regression (ENR) [45] was used as an alternative approach to select important variables associated with BMI and percent fat. ENR is similar to traditional multivariable regression models but performs variable selection: regression coefficients are shrunk towards zero, with unimportant variables given coefficients exactly equal to zero, effectively removing them from the model. Compared to other variable selection methods (e.g., least absolute shrinkage and selection operator (LASSO) [46]), ENR can better identify important variables that are correlated, which is preferable given that many variables in our study are correlated. All variables from the univariate linear regressions (excluding energy intake variables due to high missingness) were included as potential predictors (as well as sex and age), thereby allowing the model to adjust for potential confounding effects. Repeated ten-fold cross-validation (with 10 repeats) was used to tune and internally validate the prediction accuracy of the ENR models. The missForest R package [47] was used to impute any missing values in predictors, thus allowing the models to include all subjects. The outcomes were excluded from the imputation procedure in order to prevent risk of overfitting. Lastly, the variables selected by ENR were ranked from most to least important based on their “variable importance scores” using the caret R package [48].

## 3. Results

**Figure 1:**
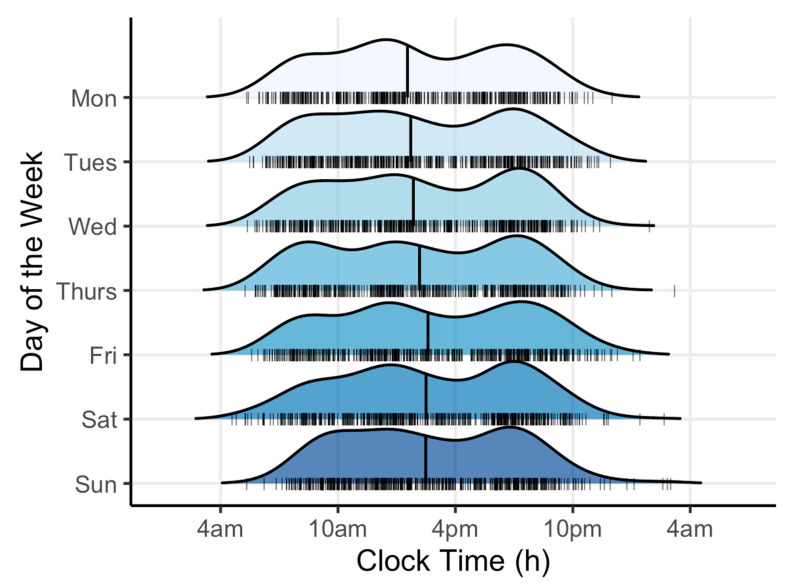
Timing of energy intake by day of the week. Ridgeline plot showing the distribution of energy intake over 24 h for each day of the recording period. Each curve shows the probability density function for energy intake with the area under each curve equal to one. The vertical lines indicate the mid-point of the eating window. The hash marks in each plot correspond to the individual photographs.

A total of 95 individuals were screened for participation in the study. Twelve were excluded (two did not meet inclusion/exclusion criteria, eight declined to participate, and two failed to complete baseline measures). Eighty-three participants were included in the final analyses, the majority of whom were females with obesity (See Table 1). Table 2 shows mean meal timing, sleep timing, physical activity and EI for all participants. Figure 1 shows the timing of EI by day of the week.

**Figure 2:**
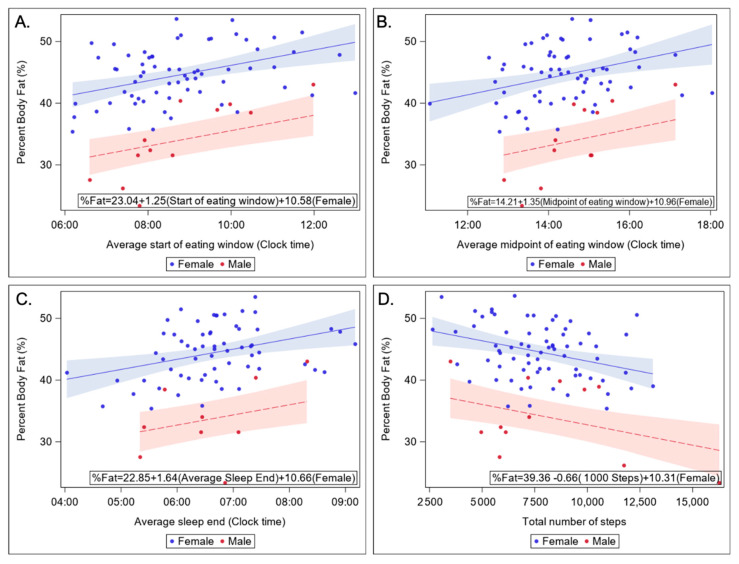
Scatter plots showing correlations between percent body fat and the start of the eating window (**A**), the mid-point of the eating window (**B**), sleep offset (**C**), and daily step counts per 1000 steps (**D**).

Age was not a significant predictor of BMI or percent fat. However, sex was a significant predictor of percent body fat. On average, females had 10.6 percent (95% CI: 7.7, 13.6) higher body fat than males. Therefore, statistical models testing significant predictors of percent body fat were adjusted for sex. Measures of meal timing (average start, midpoint, and duration), measures of physical activity (total number of steps, MET-h, and total stepping time), and measures of sleep (average sleep end and average midpoint of sleep) were significant predictors of percent fat after adjusting for sex. However, total stepping time, duration of the meal eating window, and average midpoint of sleep were not significant after the FDR adjustment (see Figure 2 and Table 3 and Table 4).

For every 1 h increase in the average start of the eating window, there was, on average, a 1.25 (95%CI: 0.60, 1.91) unit increase in percent body fat (*p*-value = 0.003, FDR *p*-value = 0.010). For every 1 h increase in the average midpoint of the eating window, there was, on average, a 1.35 (95% CI: 0.51, 2.19) unit increase in percent body fat (*p*-value = 0.002, FDR *p*-value = 0.031).

For every 1 unit increase in MET hours, there was, on average, a 1.56 (95% CI: −2.51, −0.61) unit decrease in percent body fat (*p*-value = 0.002, FDR *p*-value = 0.029). For every 1000-step increase in the total number of steps, there was, on average, a 0.66 (95% CI: −1.06, −0.26) unit decrease in percent body fat (*p*-value = 0.002, FDR *p*-value = 0.029).

For every 1 h increase in the average sleep end, there was, on average, a 1.64 (95% CI: 0.56, 2.72) unit increase in percent body fat (*p*-value = 0.003, FDR *p*-value = 0.044).

Energy intake, average start of the eating window, and the average midpoint of the eating window were significant predictors of BMI. However, start of the eating window and midpoint of the eating window were not significant after the FDR adjustment. For every 100 kcal increase in energy intake per day, there was, on average, a 0.53 (95% CI: 0.26, 79) unit increase in BMI (*p*-value = 0.0002, FDR *p* value = 0.010).

**Figure 3:**
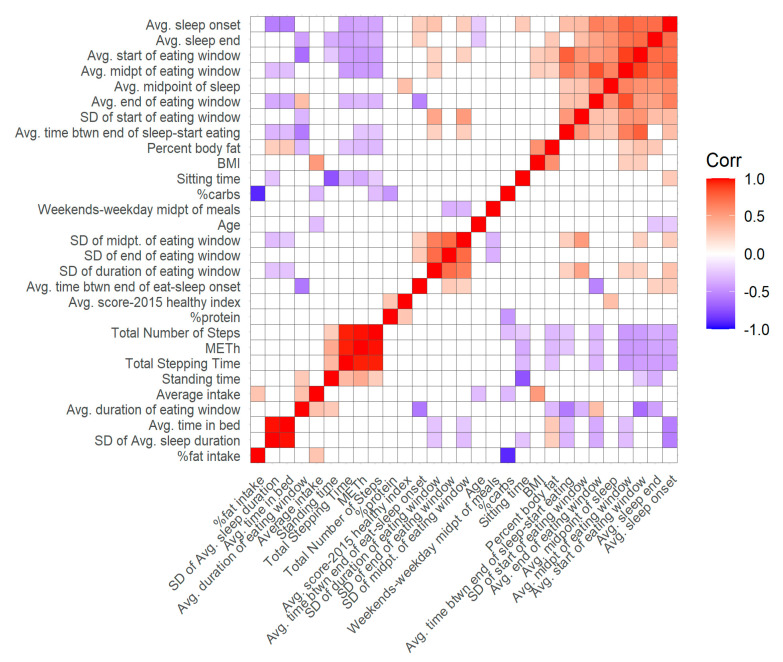
Heatmap showing correlations between body composition, energy intake, meal timing, physical activity and sleep variables. Darker shading indicates a greater degree of correlation, and correlations with *p*-values > 0.05 are displayed in white.

Figure 3 displays a Pearson’s correlation heatmap between all quantitative variables. As expected, variables measuring similar outcomes (i.e., stepping time, MET, number of steps; start, midpoint, and end of eating and sleeping windows) are highly positively correlated. Further, later start of the eating window and later end of the sleep period are associated with shorter duration of the eating window. In addition, later meal (start and midpoint of eating window) and sleep (sleep onset and sleep end) timing were negatively associated with activity levels (number of steps, stepping time, and MET-h).

**Figure 4:**
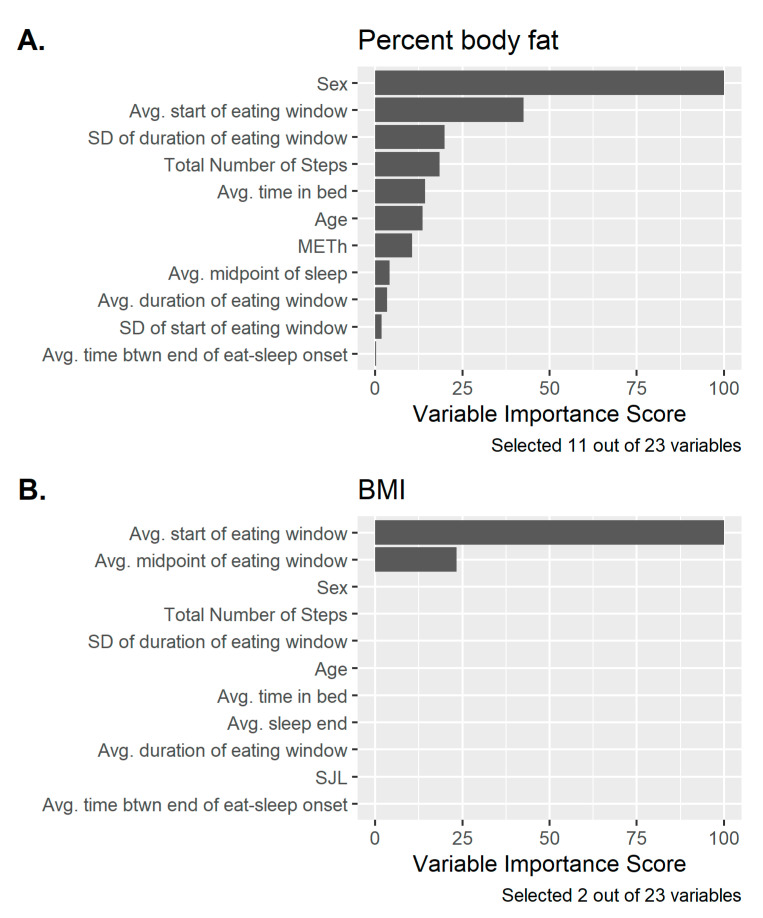
Elastic Net variable selection results showing the highest ranking meal timing, sleep, and physical activity variables as they relate to body fat percentage (**A**) and BMI (**B**).

Figure 4 displays the elastic net regression (ENR) variable selection results for both the percent body fat and BMI outcomes. Variables were sorted by their relative variable importance scores (scaled from 0 to 100, with the most important predictor having a value of 100). For percent body fat, ENR selected 11 out of 23 variables, with the top five most important predictors being sex, average start of eating window, total number of steps, standard deviation of the duration of eating window, and average time in bed. For BMI, ENR only selected two variables: average start of eating window and average midpoint of eating window. The non-zero ENR coefficients were always in the same direction as the corresponding univariate linear regression coefficients from Table 3 and Table 4, but were generally shrunk closer to zero in order to perform variable selection. Repeated 10-fold cross validation was used to assess the prediction accuracy of each model: the ENR percent fat model had a mean absolute error (MAE) of 3.9% and R2 = 43%, while the BMI ENR model had an MAE = 4.5 (kg/m^2^) and R2 = 15%.

## 4. Discussion

The main findings of our study are that later timing of EI and sleep predicted a greater percent body fat. While a few studies have shown that consuming a higher percentage of EI in the evening is associated with greater risk of obesity [26,27], these studies utilized dietary recall data, whereas we used time-stamped photographs, providing a more accurate assessment of the timing of EI. In addition, we used objective measures of physical activity and sleep. Potential mechanisms to explain the effects of meal timing on body weight and composition include effects on EI (quantity or quality of EI), physical activity levels, or metabolic effects, such as the thermic effect of food (TEF).

As expected, we found that greater total EI predicted higher BMI. However, later meal timing did not correlate with greater total EI, fat or carbohydrate intake, suggesting that the mechanism underlying the association between later meal timing and higher BMI is not due to increased EI alone. In addition, contrary to our hypothesis and to findings from other studies [14,20], we did not find that later meal or sleep timing was associated with poor dietary quality (i.e., lower Healthy Eating Index). A few studies have not shown a difference in dietary quality in later chronotypes or night shift workers [23,24,25]. Further studies are needed to better assess the relationships between meal timing and EI and diet quality.

We also found that later meal timing was associated with lower PA levels (number of steps, stepping time, and MET-h), which may represent one mechanism by which meal timing could affect weight and body fat. In the National Weight Control Registry, breakfast consumption was associated with slightly higher self-reported levels of PA among individuals who were maintaining weight loss [49], and several studies using objective measures have shown lower PA among breakfast skippers [50,51,52]. This is in contrast to a few other studies that have shown associations between later meal timing and higher BMI independent of PA levels [19,28,29]. Overall, there are limited data regarding the effects of meal timing on PA, and future studies should more carefully assess these relationships.

Our study shows that later sleep timing was associated with higher percent body fat. Several previous studies using actigraphy to measure sleep timing and duration have shown associations between later chronotype and higher BMI [3,25,37]. While two of these studies showed associations between later sleep timing and poor diet quality (higher consumption of fast food, unhealthy snacks and full-calorie soda, and fewer servings of fruits and vegetables [3,37]), the other study showed no significant differences in diet quality [25]. These studies either did not assess activity levels [25,37] or showed no difference based on sleep timing [3]. In contrast, we did not find differences in diet quality, but did show that later sleep timing was associated with lower levels of activity. In addition, our results show that later sleep timing was associated with later meal timing, so it is possible that meal timing mediates the effect of sleep timing on percent body fat. Future studies should evaluate effects of sleep timing independent of meal timing or physical activity on body weight.

We found that duration of the eating window was inversely correlated with the start of the eating window and end of the sleep period, suggesting that those who slept later started eating later and ate for a shorter period of time during the day. Shorter duration of the eating window also predicted higher percent body fat (although this relationship was not significant after FDR-adjustment), suggesting that both delaying the first meal of the day and eating over a short duration during the day were associated with higher percent body fat. These findings are consistent with prior evidence linking breakfast skipping and later timing of EI with increased risk of obesity and metabolic disease [10,11,12,13,14,15] and have important implications due to the current popularity of time-restricted eating (TRE—limiting EI to a 6–10 h window during the day) as a weight loss strategy. Several human studies have evaluated the metabolic effects of short-term, eucaloric TRE (6–8 h eating windows) aligned early in the biologic day, finding improvements in glucose homeostasis, insulin sensitivity and β cell responsiveness [53,54,55], increased fat oxidation [56], and reductions in blood pressure [54] and appetite [56]. However, most studies evaluating the effects of longer-duration TRE (8–16 weeks) on weight loss have used self-selected or mid-day eating windows [9,57,58,59,60,61] and have shown modest (2–4%) weight loss. In addition, many patients may prefer a later eating window (eating from 12–8 p.m., for example) to better align with social eating occasions [62,63], but data to support this practice are lacking. Only a few trials in humans have examined the metabolic effects of late TRE (eating windows aligned later in the day, L-TRE). In a small, 8-week, isocaloric, crossover controlled feeding trial, L-TRE (1 meal between 5–9 p.m.) as compared to three meals with no time restriction showed modest reductions in body weight and fat mass, but higher blood pressure and elevated fasting glucose levels with impaired glucose tolerance [14,64]. Resistance-trained men following L-TRE for 8 weeks (self-selected 4-h eating window between 4 p.m.–12 a.m.) significantly reduced their EI but did not exhibit changes in body composition as would be expected, suggesting that L-TRE resulted in reductions in energy expenditure or other metabolic effects that prevented them from losing weight [65]. On the other hand, Cienfuegos et al. [60] showed that both 4- and 6-h L-TRE for 8 weeks (3–7 p.m. and 1–7 p.m.) reduced weight (~3%) and improved insulin resistance and oxidative stress as compared to controls. However, the authors acknowledge that the weight loss is less than expected given the degree of caloric restriction reported (550 kcal/day) and that the improvement in insulin resistance may have been driven by worsened insulin resistance in the control arm. The largest study to date to specifically evaluate L-TRE included 116 adults with overweight and obesity randomized to either L-TRE (eating window of 12–8 p.m.) or eating three meals per day (no time restriction) for 12 weeks and showed only modest weight loss in the L-TRE group (−0.94 kg) but no significant difference between groups [66]. The lack of clear benefit from L-TRE combined with our data showing an association between higher percent body fat and later and shorter duration of eating window suggests that perhaps the observed benefits of early TRE are due more to the timing of EI as opposed to the time restriction itself. Clearly there is a need for further studies of TRE, and specifically studies evaluating the timing of TRE to determine whether later TRE results in similar metabolic benefits to early TRE.

One potential mechanism by which later meal timing might influence body weight and body composition is through effects on TEF. Several studies have shown that TEF is lower in the evening and at night than in the morning, and that these differences are explained primarily by circadian influences as opposed to behavioral effects [67,68]. Thus, eating closer to bedtime may result in a lower TEF, contributing to positive energy balance which might lead to weight gain over time. In fact, McHill et al. [19] showed that the percentage of total daily EI consumed from 4 h before dim light melatonin onset until sleep onset was significantly associated with increased body fat.

Our study is limited by the fact that these data are observational and we are reporting on correlations. Thus, we cannot definitively conclude on the causal role or directionality of the relationship between meal or sleep timing and development of overweight or obesity. In addition, our study is limited by the poor quality of EI quantification data, which were significantly limited due to the high prevalence of implausible EI (45.8%). The challenges of quantifying EI in free-living humans are well-known, and significant enough that it has been suggested that measures of self-reported EI should be abandoned [69]. Although we hypothesized that the use of photographic food records would increase the accuracy of self-reported EI, we found that omission of photographs resulted in significant under-reporting in terms of quantity of EI, while retaining important information on the timing of the eating window. While it may be argued that the high rate of implausible reports could adversely affect our measures of timing of EI, it is important to note that we counseled our participants to prioritize taking photographs of the first and last meals of the day. Underreporting is frequent when dietary recalls are used [70,71], and has also been observed in other studies utilizing photographic food records [72,73]. In order to improve the quality of our EI data, we utilized the Huang method [41] to establish cutoff values based on predicted total daily energy expenditure. While this approach results in the exclusion of a large percentage of the data and may not entirely eliminate bias [69], we believe this approach results in a more accurate representation of EI in our study sample. Future studies should employ additional measures to enhance completion of photographic dietary records such as automated text reminders. Finally, we used clock times to assess meal and sleep timing, as we were unable to obtain objective measures of chronotype (i.e., dim light melatonin onset).

## 5. Conclusions

In summary, our data demonstrate that later meal and sleep timing were associated with higher percent body fat in this sample of individuals with overweight and obesity. These findings are important, given the recent shift toward more frequent eating occasions over a longer period of time throughout the day, as well as the recent popularity of TRE using later eating windows. In addition, we found that later meal and sleep timing were associated with lower activity levels, a finding that has not previously been reported. Taken together with data from previous trials, our findings underscore the importance of the timing of daily activities in weight regulation and the need to consider the timing of EI, PA and sleep in the design and evaluation of weight loss interventions.

## Figures and Tables

**Table 1:** Demographics.

	***n* (%)**
**Sex**	
Female	71 (86%)
Male	12 (14%)
**Race**	
American Indian/Alaska Native	0 (0%)
Asian	5 (6%)
Native Hawaiian or Other Pacific Islander	0 (0%)
Black or African American	10 (12%)
White	67 (81%)
More than one race	0 (0%)
Unknown/not reported	1 (1%)
**Ethnicity**	
Hispanic or Latino	13 (16%)
Not Hispanic or Latino	69 (83%)
Unknown/not reported	1 (1%)
	**Mean (Std Dev)**
Age (years)	38.7 (7.8)
Weight (kg)	93.8 (17.8)
BMI (kg/m^2^)	33.7 (5.6)
Lean body mass (kg)	53.0 (9.4)
Fat mass (kg)	40.8 (11.5)
Percent fat (%)	43.1 (6)

**Table 2:** Meal timing, sleep timing, physical activity and energy intake.

Meal Timing	*n*	Mean (SD)
Start of eating window (clock time)	77	08:48 (01:30)
Standard deviation of start of eating window (hours:mins)	77	01:18 (00:42)
Midpoint of eating window (clock time)	77	14:30 (01:12)
Standard deviation of midpoint of eating window (hours:mins)	77	1:06 (00:30)
End of eating window (clock time)	77	20:06 (01:18)
Standard deviation of end of eating window (hours:mins)	77	01:36 (00:48)
Duration of eating window (hours:mins)	77	11:18 (01:24)
Standard deviation of duration of eating window (hours:mins)	77	02:12 (02:00)
Midpoint of meal timing on weekends minus weekdays (hours:mins)	66	−00:36 (01:00)
**Sleep**		
Social Jet Lag (sleep timing on weekends minus weekdays) (hours:mins)	71	00:54 (01:24)
Time of sleep onset (clock time)	71	23:18 (01:12)
Time of sleep offset (clock time)	71	06:36 (01:00)
Time in bed (hours:mins)	71	07:18 (00:48)
Sleep duration (hours:mins)	71	06:48 (00:48)
Midpoint of sleep (clock time)	71	03:06 (01:06)
**Sleep and Meal timing**		
Time elapsed between sleep offset and start of eating window (hours:mins)	66	02:12 (01:06)
Time elapsed between end of eating window and sleep onset (hours:mins)	66	03:12 (01:00)
**Primary Physical Activity**		
Stepping time (min)	82	95.7 (27.9)
Steps (n)	82	7769.0 (2473.5)
Standing time (min)	82	222.3 (76.9)
Sitting time (min)	82	630.2 (90.4)
Metabolic Equivalents (METh)	82	33.6 (1.0)
**Energy intake**		
Fat (%)	45	38.1 (6.3)
Carbohydrate (%)	45	45.2 (7.1)
Protein (%)	45	16.6 (3.1)
Intake (kcals)	45	2328.6 (481.8)
Healthy Eating Index Score	45	55.1 (12.5)

**Table 3:** Linear regressions with percent body fat.

	Estimate [95% CI]	Raw *p*-Value	FDR-Adjusted *p*-Value	*n*
Start of eating window (hour)	1.25 [0.6, 1.91]	0.0003	**0.010**	77
MET-h	−1.57 [−2.52, −0.62]	0.002	**0.029**	82
Total number of steps (per 1000 steps)	−0.66 [−1.06, −0.26]	0.002	**0.029**	82
Midpoint of eating window (hour)	1.35 [0.51, 2.19]	0.002	**0.031**	77
Time of sleep offset (hour)	1.64 [0.56, 2.72]	0.003	**0.044**	71
Total stepping time (hour)	−3.02 [−5.21, −0.83]	0.008	0.083	82
Duration of eating window (hour)	−0.96 [−1.71, −0.21]	0.013	0.117	77
Midpoint of sleep (hour)	1.12 [0.13, 2.11]	0.028	0.193	71
Time of sleep onset (hour)	0.95 [−0.01, 1.91]	0.053	0.346	71
Standing time (hour)	−0.68 [−1.49, 0.14]	0.102	0.413	82
End of eating window (hour)	0.67 [−0.18, 1.52]	0.123	0.413	77
Standard deviation of start of eating window (hour)	1.11 [−0.34, 2.56]	0.133	0.413	77
Standard deviation of end of eating window (hour)	−0.85 [−2.16, 0.47]	0.205	0.440	77
Time elapsed between sleep offset and start of eating window (hour)	0.60 [−0.41, 1.6]	0.238	0.476	66
Standard deviation of duration of eating window (hour)	−0.66 [−1.91, 0.58]	0.292	0.519	77
Protein intake (%)	0.19 [−0.22, 0.6]	0.361	0.577	45
Sitting time (hour)	0.30 [−0.41, 1.01]	0.399	0.620	82
Time in bed (hour)	0.60 [−0.88, 2.07]	0.423	0.647	71
Time elapsed between end of eating window and sleep onset (hour)	−0.41 [−1.51, 0.69]	0.458	0.684	66
Standard deviation of average sleep duration (hour)	0.35 [−1.23, 1.94]	0.658	0.856	71
Carbohydrate intake (%)	−0.04 [−0.21, 0.14]	0.676	0.858	45
Energy intake (100 kcals)	0.02 [−0.24, 0.29]	0.870	0.973	45
Metabolic jetlag (midpoint of meal timing weekends-weekdays) (hour)	0.05 [−1.08, 1.17]	0.934	0.985	66
Standard deviation of midpoint of eating window (hours)	−0.060 [−2.28, 2.16]	0.958	0.985	77
2015 healthy index score	−0.002 [−0.11, 0.1]	0.974	0.985	45
Fat intake (%)	0.003 [−0.2, 0.2]	0.976	0.985	45

Percent body fat models were adjusted for sex. MET-h: metabolic equivalents per hour. Variables for which FDR adjusted *p*-values are < 0.05 are bolded.

**Table 4:** Linear regressions with body mass index (BMI).

	Estimate [95% CI]	Raw *p*-Value	FDR-Adjusted *p*-Value	*n*
Energy intake (100 kcals)	0.53 [0.26, 0.79]	0.0002	**0.010**	45
Start of eating window (hour)	0.98 [0.19, 1.77]	0.016	0.130	77
Midpoint of eating window (hour)	1.16 [0.16, 2.16]	0.023	0.172	77
Time of sleep offset (hour)	1.06 [−0.29, 2.4]	0.123	0.413	71
Midpoint of sleep (hour)	0.92 [−0.29, 2.12]	0.134	0.413	71
Carbohydrate intake (%)	−0.15 [−0.36, 0.05]	0.140	0.413	45
Time elapsed between sleep offset and start of eating window (hour)	0.93 [−0.32, 2.18]	0.141	0.413	66
End of eating window (hour)	0.72 [−0.26, 1.69]	0.147	0.413	77
Standard deviation of start of eating window (hour)	1.17 [−0.51, 2.84]	0.169	0.440	77
Total number of steps (per 1000 steps)	−0.32 [−0.82, 0.18]	0.207	0.440	82
Duration of eating window (hour)	−0.56 [−1.45, 0.32]	0.207	0.440	77
Time of sleep onset (hour)	0.70 [−0.44, 1.84]	0.224	0.457	71
Fat intake (%)	0.14 [−0.1, 0.37]	0.249	0.480	45
MET-h	−0.63 [−1.81, 0.54]	0.286	0.519	82
Metabolic jetlag (midpoint of meal timing weekends-weekdays) (hour)	0.69 [−0.62, 2.01]	0.294	0.519	66
Protein intake (%)	0.24 [−0.23, 0.72]	0.308	0.534	45
2015 healthy index score	−0.06 [−0.18, 0.06]	0.324	0.553	45
Standard deviation of midpoint of eating window (hour)	1.24 [−1.3, 3.77]	0.334	0.560	77
Stepping time (hour)	−1.15 [−3.82, 1.51]	0.393	0.609	82
Sitting time (hour)	0.24 [−0.58, 1.07]	0.557	0.772	82
Standing time (hour)	0.17 [−0.8, 1.14]	0.723	0.894	82
Sleep duration (hour)	−0.31 [−2.08, 1.46]	0.730	0.894	71
Time elapsed between end of eating window and sleep onset (hour)	−0.10 [−1.48, 1.27]	0.879	0.973	66
Time in bed (hour)	0.09 [−1.57, 1.75]	0.913	0.985	71
Standard deviation of duration of eating window (hour)	0.03 [−1.41, 1.47]	0.964	0.985	77
Standard deviation of end of eating window (hour)	0.003 [−1.53, 1.54]	0.997	0.997	77

Variables for which FDR adjusted *p*-values are < 0.05 are bolded.

## Data Availability

Data is available for research purpose upon reasonable request to the corresponding author.
